# Intelligence

**Published:** 1809-06

**Authors:** 


### INTELLIGENCE.

The following Cautions have been recommended by the Physicians and Surgeons of the Bath Hospital, to those who have received benefit by the use of the Bath Waters, in cases where the poison of lead is concerned, as plumbers, glaziers, painters, and other artificers, who work in trades which expose them to similar hazards from the same cause; to be observed by them at their return to the exercise of their former occupations:—

"To maintain the strictest temperance, particularly respecting distilled spirits, which had better be altogether forborne.—To pay the strictest attention to cleanliness; and never, when it can be avoided, to daub their hands with paint; and particularly never to eat their meals, or go to rest, without washing their hands and face.—Not to eat or drink in the room or place wherein they work, and much less to suffer any food or drink to remain exposed to the fumes or dust of the metal, in the workshops or warehouses.—As the clothes of persons in this line (painters particularly) are generally observed to be much soiled with the colours they use, it is recommended to them to perform their work in frocks or ticken, which may be frequently washed, and conveniently laid aside, when the workmen go to their meals; and again put on when they resume their work. Every business which can, in these branches, should be performed with gloves on the hands, and woollen or worsted gloves are recommended, as they may be often washed, as they should always be after being soiled with paint, or even by rubbing against the metal.—Caution is necessary in mixing, or even in unpacking, the dry colours, that the fine powder does not get into their mouths, or be drawn in by the breath. A crape covering over the face might be of service, but care should be taken to turn always the same side towards the face, and to clean or wash it frequently. All artificers should avoid touching lead when hot; and this caution is especially necessary for printers or compositors, who have often lost the use of their limbs by handling the types when drying by the fire, after being washed. Glaziers' putty should never be made or moulded by the hand. An iron pestle and mortar would work the ingredients together, at least equally well, and without hazard.—If any person, in any of the above employments, should feel pain in the bowels, with costiveness, they should immediately take twenty drops of laudanum, and when the pain is abated, two table spoonsful of Castor oil, or an ounce of the bitter purging salt, dissolved in warm chamomile tea. If this does not succeed, a pint or two pints of warm soap-suds should be thrown up as a clyster.—As a preventive, two or three tea-spoonsful of sallad-oil, taken in a small cup of gruel, are likely to be of service, if taken daily, and steadily pursued."

The

The perusal of the Report by Messrs. Fourcroy, Deyeux, and Vauquelin, on a Memoir of M. Bertholet, jun. entitled, "Inquiries concerning the reciprocal Action of Sulphur and Charcoal," has induced Dr. John New to publish an opinion, which he has for some years entertained, — that charcoal and hydrogen are modifications of one and the same substance, or that hydrogen is the base of charcoal. Should this opinion, the result of various experiments and observations, be confirmed, an important and extensive field will be opened to the scientific world. The pabulum of plants, and the origin of that immense quantity of carbonaceous matter, annually produced in the vegetable kingdom, would thus easily and satisfactorily be accounted for, as originating from water alone.

Professor Davy has now completed his grand galvanic battery, on a plan principally his own. In pursuing his experiments in the public lecture room, he made a most beautiful exhibition of the matter of fire, if we may use so strong an expression. By uniting the two ends of the battery with the medium of only a thin wire, that wire instantly became red, and afterwards white hot, and remained so as long as the communication continued. This was enough to show that the electric fire is not the mere effect of the sudden communication between positive and negative electricity.— He afterwards repeated the experiment in a glass exhausted receiver. The same phenomena appeared. By this it follows, that the matter of fire may be visible for any length of time in a vacuum, consequently, that its appearance does not in all cases depend on a chemical decomposition of the air. In his last lecture, after expressing himself with more feeling than was necessary on the premature inquiries of some concerning the probable uses of his newly discovered metals, he produced a specimen of the wootz, a substance resembling steel, brought from the East Indies some years ago, and analyzed by Dr. Pearson; an account of which was read before the Royal Society in the year 1795, and published in their Transactions. In this paper Dr. Pearson remarks, that he detected silex in the wootz; but considers it only an adventitious substance, forming no necessary part of that metal. Professor Davy, on the contrary, finds that the metal which forms the basis of silex, is intimately mixed with wootz, and is probably the cause of all those properties, in which it differs from common steel. The metal of silex, like all the others discovered by the Professor, is so greedy of oxygen, that in every experiment it will elude the research without great care, becoming an oxyd, and exhibiting no other appearance than silex.

Mr. Taunton, will, on Saturday June the 3d, 1809, commence his Summer Course of Lectures on Anatomy, Physiology, Pathology, and Surgery, at Eight o'Clock in the Evening, *precisely*, which will be continued every Tuesday, Thursday, and Saturday, at the same hour.—Particulars may be had on applying to Mr. Taunton, Greville-Street, Hatton-Garden.

Mr.

Mr. J. WILSON, Surgeon, late of Guy's Hospital, will publish in the course of next month, PHARMACOPEA CHIRURGICA, or a Formulæ of the different Hospitals.

Dr. SERNY, Oculist, has in the press, and will speedily publish, A Treatise on local Inflammation, more particularly applied to Diseases of the Eye; wherein an improvement in the treatment of those diseases is recommended, which has been confirmed by numerous cases under the Author's own care.

